# Progress in Tropical Diseases

**Published:** 1902-12-27

**Authors:** 


### PROGRESS IN TROPICAL DISEASES.

(*Concluded from page* 198.)

**Dysentery.**—A. Duncan [2] deals with dysentery as it occurs in India. That the term dysentery as denoting a disease of which the main symptoms are pain, tenesmus, and the passage of blood from the bowel, includes more than one disease, he holds is proved by the bacteriological findings and by the results of treatment. Councilman and Lafleur have spoken of amœbic dysentery as the form commonly called tropical, and certain distinguishing clinical features have been claimed for this form. Among these are its chronicity, liability to relapse, and to the formation of liver abscess, its extreme fatality, low grade of fever, and the evacuation of fluid stools containing amœbæ, also the less frequent occurrence of pain and tenesmus. Although India is a tropical country, Duncan holds that the amœba has nothing to do with the Indian type of dysentery. He has found the amœba as frequently absent as present in the stools, has noticed no marked diminution of pain and tenesmus, thinks that fever is always mild in this disease, and thinks that the prognosis is very favourable if the disease is treated early. Chronic cases he finds extremely rare, and although multiple liver abscesses are related to dysentery, the large tropical abscess is denied any special relation to the disease. Unfavourable signs in prognosis are, persistence of blood and mucus, and the occasional passage of fluid motions or of fœtid porridge-like stools. Blackish-red, fluid, horribly putrescent stools, mixed with shreds of gangrenous tissue, indicate a fatal issue. For treatment of the acute disease this observer has found ipecacuanha invariably well borne by natives and very efficacious. Intolerance is sometimes met with among Europeans when an excellent substitute is found in cinnamon, a drachm being given night and morning. In Africa ipecacuhana seems to fail, and salines are more beneficial. Lavage with boric acid seems successful in Malay, while during an outbreak in Grenada sulphur and Dover's powder gave the best results. Buchanan records his experience in the prevention and treatment of dysentery in Indian prisons. He urges the communicability of the disease, and insists on isolation, rigid disinfection of clothes, bedding, etc., and disinfection and incineration of the stools, together with their protection from flies. He has frequently observed outbreaks to follow the issue of badly cooked, ill-cleaned grain food such as rice or wheat to the prisoners. Early and thorough treatment carried out in a hospital till the patient is really cured will best prevent relapses, and relapsed dysentery is frequently fatal. In the treatment of acute cases rest in bed and milk diet at first are essential, while for drugs nothing has been found by this observer equal to salines. Sodium sulphate is preferred, given in drachm doses four, five, or six times a day in cinnamon or fennel water. The administration is continued until bright yellow, soft, fæculent stools are being passed, without a trace of pain or blood or mucus. In the treatment in this way of 1,130 cases, only 9 deaths occurred, a mortality of under 1 per cent. Buchanan considers that amœbic dysentery, though rare, does occur in India, but that most cases are of bacillary origin, due to the presence of Shiga's bacillus. L. Rogers, as the result of his studies and investigations in Calcutta, finds a very close relation between the amœbic form of dysentery and the production of liver abscess. The idea that liver abscess is rare among the natives of India is considered quite erroneous. The amœba is not always found in the pus of liver abscess, but, excluding two cases not examined until 12 or 14 days after the abscess had been opened, in 35 consecutive cases amœbæ were readily found in scrapings from the wall of the abscess cavity. Pyogenic organisms were not present in the pus in the majority of cases, and no abscess formed on injecting a cat with pus from one case containing amœbæ and staphylococci. In the majority of the cases of "tropical" liver abscess, that is of large single or multiple abscess, not small multiple pyæmic abscesses, of which the clinical and post-mortem records were examined, there was a history of previous dysentery, and dysenteric lesions were found in the large bowel. In other cases lesions—of the amœbic variety—were found post-mortem when the clinical history was wanting, while in a third series of cases which had clinically suffered from dysentery no post-mortem lesions were found. Of 63 cases with complete clinical or post-mortem records evidence, clinical, post-mortem, or both of previous dysentery was obtained in 90·48 per cent. Of 40 cases 95 per cent. were found to give positive evidenc of dysentery preceding the formation of the liver abscess, as shown by the onset of fever and hepatic pain. In a series of eight cases examined, in which the amœba was sought for in the intestinal ulcerations, in each it was readily found in the yellow material in the bases of the ulcers. From this and other evidence adduced the conclusion is reached that the form of dysentery found associated with tropical liver abscess is that due to the amœba dysenterica. Amœbic dysentery is rare in India, the vast majority of cases being of the ordinary catarrhal type which is being traced with increasing frequency to the bacillus of Shiga. If, therefore, the above view of the relation of tropical liver abscess to amœbic dysentry be correct it will explain the rarity of the occurrence of tropical abscess among armies in the field and in Indian gaols and European asylums. The naked eye features of the intestinal ulcerations met with in amœbic dysentery are described. The ulcers vary in size from a big pin's head to an area of several square inches, and consist generally of raised yellowish gelatinous patches marked off sharply by a narrow zone of intense congestion from the surrounding healthy mucous membrane. The raised character of the ulcers and the absence of thickening and congestion of the non-ulcerated portions of the bowel distinguish this form of ulceration from that met with in ordinary cases. The largest ulcers are met with in the cæcum and the ascending colon, but the disease never in this variety extends above the iliocæcal valve. The liver abscess, even when small, is rarely situated deep in its substance; it is found just under the surface in the neighbourhood of the suspensory ligament. Great stress is laid on the value of leucocytosis as a diagnostic feature in liver abscess, especially in helping to distinguish it from some forms of malaria. The introduction of concentrated solutions of quinine, 1 in 100 or 1 in 80, into the incised or aspirated abscess cavity is recommended. Outside the body the amœbæ are rapidly killed by such solutions.

[2] Brit. Med. Jour., Sept. 20.

